# Effect of 0.2% peracetic acid disinfection on the horizontal dimension of implant framework indexed with two autopolymerized acrylic resins

**DOI:** 10.1186/s40729-019-0186-2

**Published:** 2019-09-18

**Authors:** Emanuely Ronconi da Fonseca, Paula Pereira Santana, Anuar Antonio Xible, Donald A. Curtis, Stefania Carvalho Kano

**Affiliations:** 1 Clinical Dentistry Master Program, Universidade Federal do Espiríto Santo, Av. Marechal Campos, 1468 – Maruípe, Vitória, ES 29040-090 Brazil; 2 Department of Prosthodontic, Universidade Federal do Espírito Santo, Av. Marechal Campos, 1468 – Maruípe, Vitória, ES 29040-090 Brazil; 3 Department of Preventive and Restorative Dental Sciences, School of Dentistry, University of California, San Francisco, 707 Parnassus Avenue, San Francisco, CA 94143-0758 USA

**Keywords:** Dental implants, Disinfection, Prosthesis fitting, Peracetic acid

## Abstract

**Background:**

To minimize misfit between prosthesis and implant, the welding of the implant fixed partial denture (IFPD) framework is recommended and autopolymerized acrylic resin (AR) is the material of choice for the indexing process. As for any prosthetic device that comes into contact with saliva and blood in the oral cavity, IFPD indexed with AR must be disinfected before sending to the laboratory. However, disinfection procedures are often neglected for fear of shrinkage or distortion caused by a dimensional change of the acrylic resin. Peracetic acid is a high-level disinfectant agent at low concentrations and immersion time, with no reported toxic residues, and it is not inactivated in the presence of organic matter when compared to other disinfectants. This study aimed to evaluate the influence of 0.2% peracetic acid disinfectant solution after different storage media and times on the horizontal dimension of IFPD frameworks indexed with AR.

**Material and methods:**

IFPD frameworks were indexed with two AR: group 1 Duralay and group 2 Pattern Resin LS. Each group was further divided into five subgroups according to disinfection procedure and storage medium: no disinfection and dry storage, no disinfection and water storage, 0.2% peracetic acid disinfection and water storage, 0.2% peracetic acid disinfection and peracetic acid storage, and 0.2% peracetic acid disinfection and dry storage. The horizontal dimension of the specimens and an average was established for analysis. Measurements were performed at four different storage times (hours): T0, T24, T48, T168.

**Results:**

No statistical differences were found when T0 was compared to T168 for Pattern resin groups submitted to disinfection and storage in water (group 2b, *p* = 1.000) or peracetic acid solution (group 2c, *p* = 0.352). For Duralay groups, the use of peracetic acid solution did not affect the horizontal dimension of the specimens when T0 was compared to T168 only with water as a storage medium (group 1b, *p* = 1.000). Additionally, T0 did not differ from T24 for groups 1c (*p* = 0.553), 2b (*p* = 1.000), 2d (*p* = 0.234), and 2e (*p* = 1.000) and from T48 for groups 1d (*p* = 0.118) and 2b (*p* = 1.000).

**Conclusion:**

Within the studied conditions, the use of 0.2% peracetic acid can be safely used as a disinfectant solution regarding dimensional stability of AR-indexed IFPD until 7 days of storage. Horizontal discrepancies are dependent on acrylic resin type, time, and medium of storage.

## Background

The long-term outcome of treatment with an implant may be compromised by the lack of adaptation between prosthesis and implant and can result in prosthesis failure, buildup of bacteria in soft and hard tissue, reactions as mucositis and periimplantites, and even loss of osseointegration [1–4]. As the several steps involving the fabrication of the prostheses can cause distortion, the welding of the implant fixed partial denture framework (IFPD) is recommended to minimize misfit between prosthesis and implant [5–7]. According to Branemark [8], misfits at the prosthesis level of up to 10 μm can be biomechanically accepted and is considered a passive fit prosthesis. Although vertical discrepancies are commonly used for implant misfit analysis, horizontal misfits can also be harmful and should be used to evaluate framework fabrication techniques, since linear distortion can be created by contraction during the fabrication process of the prostheses [9]. When conventional welding is to be used, autopolymerized acrylic resin (AR) is the material of choice for the indexing process as described by Patterson [10], and the behavior of the material used to index the parts to be welded should be known [11].

As for any prosthetic device that contacts saliva and blood in the oral cavity, the IFPD joined with AR may become a vehicle for cross-contamination [12], therefore it should be cleaned and disinfected before being sent to the laboratory. Heat-sensitive critical and semi-critical instruments and devices can be sterilized by immersion in liquid chemical germicides having at least an intermediate level of activity [13].

Among the disinfectant solutions available, 2% glutaraldehyde and 1% sodium hypochlorite have been widely used, and both are linked with a variety of health effects and require longer immersion time when compared to peracetic acid. Additionally, both are inactivated by organic matter [13–16].

Peracetic acid is a liquid chemical germicide listed as a sterilant and high-level disinfection product that has been used for medical and dental purposes [16–20]. The increased interest on peracetic acid is due to its effective broad-spectrum disinfection properties with bactericidal, virucidal, and sporicidal effect and no reported toxic residues [17]. Peracetic acid acts rapidly against all microorganisms even at low concentrations and low immersion time and is not inactivated in the presence of organic matter when compared to other disinfectants such as sodium hypochlorite and glutaraldehyde [13, 17]. There is also a concern about the effect of these disinfectants on acrylic resin properties [17].

Autopolymerized acrylic resin presents polymerization shrinkage, which it may be influenced by storage time and medium [21–25], suggesting that indexed prostheses should be sent as soon as possible for laboratory processing to minimize distortion. Usually, the period from clinical indexing procedure and laboratory processing takes 24 or more hours to be performed and can take longer if dental clinics and laboratory are not located in the same area.

The main objective of this work was to evaluate the horizontal dimensional stability of IFPcection with 0.2% peracetic acid solution and after different media and times of storage.

## Material and methods

### Specimen fabrication

**Fig. 1:**
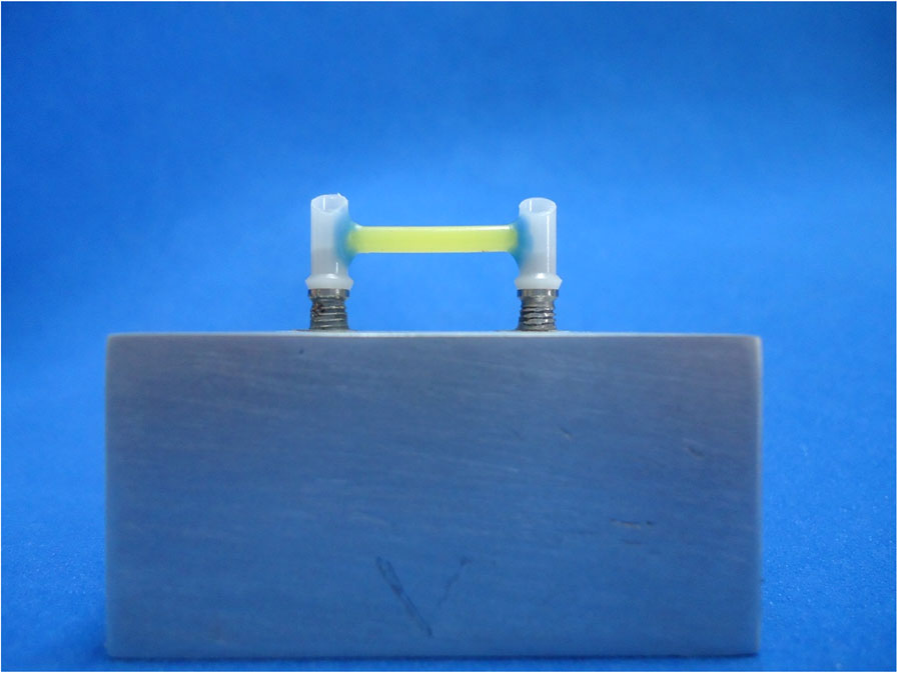
UCLA casting cylinders attached with wax bar before casting

On a master template with two external hexagon-type implants of 3.75 mm and 4.1 mm platform (Neodent, Curitiba, Paraná, Brazil), metal IFPD infrastructures were fabricated using hexagonal UCLA casting cylinders (Neodent, Curitiba, Paraná, Brazil) attached by 2.5 mm in diameter cylindrical prefabricated wax bar (Ceras Babinete Ltda, Maringá, Paraná, Brazil) positioned 4 mm above the base (Fig. 1).

Waxed specimens were included and cast with CoCr alloy (CoCr DeguDent, Dentsply, São Paulo, Brazil) by centrifugal technique, following the manufacturer’s recommendations. After casting, specimens were sandblasted and internal surfaces were inspected with a 3.5× magnifier (Bio-Art, São Carlos, São Paulo, Brazil). Each specimen was then sectioned with an aluminum oxide disc (Dentorium International Incorporated, New York, USA) with an approximate thickness of 0.3 mm.

### Indexing process

**Fig. 2:**
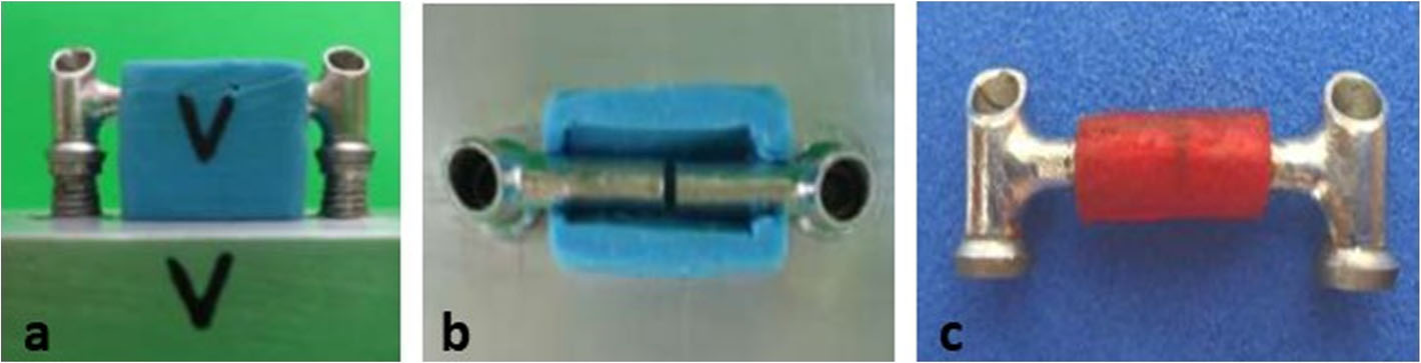
**a**, **b** Silicon model to standardize resin volume. **c** IFPD indexed with acrylic resin

Specimens were manually screwed in place and 32 Ncm torque was applied to the screws with a torque wrench (Ratchet Wrench, Neodent, Curitiba, Brazil), indexed with AR, and allowed to cure for 10 min. During the indexing procedure, autopolymerized acrylic resin was inserted with the aid of a brush using the technique described by Nealon [26] and a silicon model to standardize AR volume (Fig. 2).

### Measurement of the horizontal dimension of the specimen

**Fig. 3:**
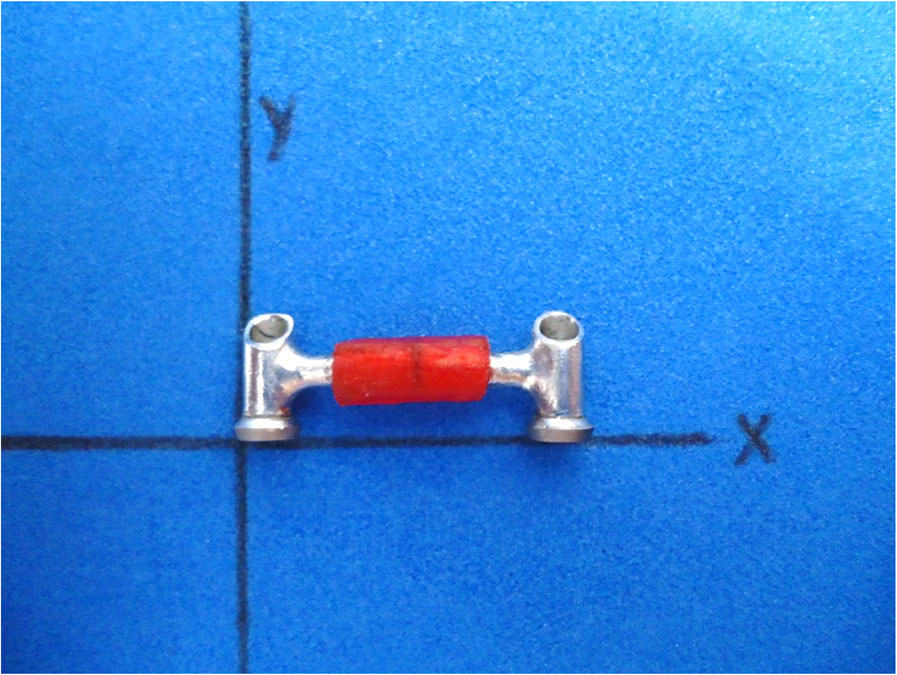
Horizontal dimension considering the length between the most distal prosthetic surfaces of the framework

All IFPD frameworks were unscrewed from the master model (Fig. 2c) and were taken to the video optical measuring instrument for image processing (Tesa Visio 200 model, Renes, Switzerland) with an accuracy of 1 μm, to measure horizontal dimension alteration. The horizontal dimension was the total length between the abutments at the most distal prosthetic surface of the IFPD on the *x*-axis of the machine and was completed with transmitted light illumination (Fig. 3).

The first measurement was performed 10 min after polymerization of AR (T0). To evaluate the effect of storage time and storage medium on the horizontal dimension of the AR, additional measurements were taken at three different moments (Table 1): T24, 24 h of storage; T48, 48 h of storage; and T168, 168 h or 7 days of storage.

**Table 1:** Description of storage time

Storage time	Description
T0	Initial measurement
T24	24 h of storage
T48	48 h of storage
T168	168 h or 7 days of storage

Each specimen at each moment of analysis was measured three times, and a mean horizontal dimension (millimeters) of each specimen was obtained. All measurements were completed by a single clinician who was blinded to both the disinfection/storage procedure and type of AR.

During the whole experimental period, specimens were stored in controlled room temperature (20 ± 2 °C) and 50% relative air humidity.

### Disinfection/storage procedures and specimen grouping

The specimens were aleatorily divided into two groups according to the AR used: group 1 Duralay (Reliance Dental Mfg. Co., IL, USA) and group 2 Pattern Resin LS (GC AMERICA Inc., IL, USA). Further, each group was divided into five subgroups with 16 specimens each (*n* = 16) according to disinfection/storage condition (Table 2).

**Table 2:** Group description according to disinfection procedure and storage medium (*n* = 160)

Description	Group 1 (Duralay) (*n* = 80)	Group 2 (Pattern) (*n* = 80)
No disinfection, water storage	1a	2a
Disinfection in 0.2% peracetic acid for 10 min and water storage	1b	2b
Disinfection in 0.2% peracetic acid for 10 min and peracetic acid storage	1c	2c
Disinfection in 0.2% peracetic acid for 10 min and dry storage	1d	2d
No disinfection, dry storage	1e	2e

Groups 1a and 2a were not submitted to the disinfection procedure and were stored in water. Groups 1e and 2e were not submitted to the disinfection procedure and were stored in dry condition. Groups 1b, 2b, 1c, 2c, 1d, and 2d were submitted to the same disinfection protocol but different storage conditions after disinfection. Groups 1b and 2b were stored in water, groups 1c and 2c were stored in 0.2% peracetic acid, and groups 1d and 2d were stored in dry condition.

The disinfection protocol used was a high-level disinfection according to the manufacturer’s recommendation and consisted of a 10 min immersion in 0.2% peracetic acid (PERAX RIO, Rioquímica, São José do Rio Preto, São Paulo, Brazil), followed by rinsing in physiological solution of 0.9% sodium chloride (Arboreto, Juiz de Fora, Minas Gerais, Brazil).

Groups 1a, 2a, 1e, and 2e were not submitted to a disinfection procedure and were used as a control group to compare the horizontal dimensional change when specimens are submitted to disinfection procedure with 0.2% peracetic acid (groups 1b, 2b, 1c, 2c, 1d, and 2d).

Data were treated with the statistical program SPSS 24 (IBM, Armonk, NY, USA) with repeated measures ANOVA and differences established with a Bonferroni test. The significance level adopted was 5%.

## Results

The results for the horizontal dimension (mean and standard deviation) for all groups according to the time of storage are presented in Table 3. Post hoc Bonferroni test was used to detect differences between groups.

**Table 3:** Mean values and standard deviation of the horizontal dimension of the specimens (millimeters) for all groups at all storage time (*n* = 160)

Group	Storage time	Mean	Standard deviation (SD)	*p*
1a	T0	25,511.48^c^	70.09	**< 0.001**
	T24	25,506.52^ab^	71.44	
	T48	25,504.1^a^	70.61	
	T168	25,507.4^b^	70.63	
1b	T0	25,525.54^b^	50.6	**< 0.001**
	T24	25,518.87^a^	50.28	
	T48	25,518.29^a^	50.36	
	T168	25,524.9^b^	50.24	
1c	T0	25,536.9^c^	45.64	**< 0.001**
	T24	25,534.88^bc^	42.95	
	T48	25,530.58^a^	43.58	
	T168	25,531.6^ab^	45.28	
1d	T0	25,519.36^b^	40.44	**< 0.001**
	T24	25,512.52^a^	40.33	
	T48	25,515.35^ab^	42.55	
	T168	25,513.4^a^	41.76	
1e	T0	25,532.69^b^	52.65	**< 0.001**
	T24	25,523.33^a^	57.56	
	T48	25,525.69^a^	55.83	
	T168	25,524.25^a^	56.11	
2a	T0	25,536.71^b^	46.65	**< 0.001**
	T24	25,531.31^a^	46.4	
	T48	25,532.81^a^	47.22	
	T168	25,533.94^ab^	45.72	
2b	T0	25,541.44	48.77	0.598
	T24	25,536.71	49.02	
	T48	25,537.29	50.17	
	T168	25,538.21	52.27	
2c	T0	25,483.96^b^	85.47	**0.001**
	T24	25,479.67^a^	85.96	
	T48	25,477.58^a^	86.96	
	T168	25,480.85^ab^	87.27	
2d	T0	25,557.98^b^	63.04	**0.003**
	T24	25,554.98^ab^	62.15	
	T48	25,552.85^c^	62.11	
	T168	25,555.21^ac^	63.04	
2e	T0	25,538.73^b^	56.23	**0.005**
	T24	25,536.98^ab^	55.96	
	T48	25,533.42^a^	54.06	
	T168	25,535.23^ab^	53.69	

Different letters (abc) indicate significant differences between means (Bonferroni multiple comparison test)

Significant level for Repeated measures ANOVA were in bold

The use of 0.2% peracetic acid as disinfectant did not affect the horizontal dimension of Pattern resin groups when T0 was compared to T168 if specimens were not stored in dry medium (group 2b, *p* = 1.000; group 2c, *p* = 0.352). The same was true for Duralay resin groups only when stored in water (group 1b, *p* = 1.000). The use of 0.2% peracetic acid as disinfectant followed by water storage did not alter the horizontal dimension of Pattern resin (group 2b) when T0 was compared to all storage times (T24, T48, T168) (*p* = 1.000).

**Fig. 4:**
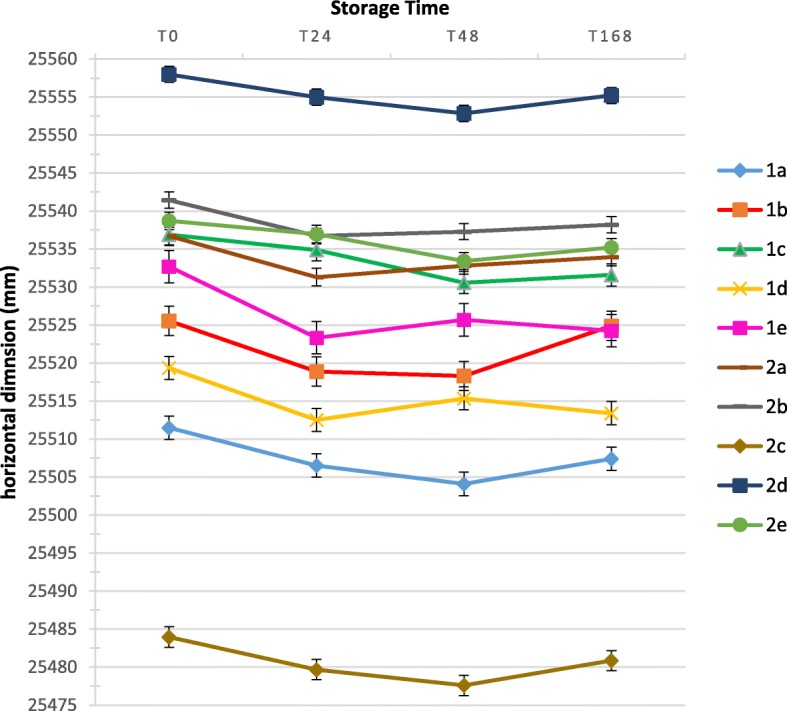
Horizontal dimension for all groups analyzed at all storage times

After disinfection, horizontal contraction was observed after 24 h (T24) for groups 2c, 1b, and 1d and after 48 h (T48) for groups 2c, 2d, 1b, and 1c (*p* ≤ 0.05). When comparing storage medium (dry × water) and no disinfection, Duralay resin groups (1a and 1e) presented a statistically significant reduction of the horizontal dimension when T0 was compared to all storage times (T24, T48, and T168). For Pattern resin groups (2a and 2e), when T0 was compared to T168, no differences were found for group 2a (*p =* 0.053) and 2e (*p* = 0.208) and also when T0 was compared to T24 for group 2e (*p* = 1.000). Figure 4 shows the variance of horizontal dimension for all groups analyzed.

## Discussion

The guidance of the American Dental Association and the Center for Disease Control and Prevention recommend that all denture devices must be disinfected before they are sent to the laboratory technician to eliminate cross-contamination [27]. In this study, we used 0.2% peracetic acid for disinfection of the specimens and as storage medium after indexing the IFPDs with acrylic resin, to assess whether peracetic acid as a disinfection product and as a storage medium would cause horizontal dimensional changes on the acrylic resin used to index IFPDs for soldering.

The use of peracetic acid on microbiological disinfection was reported previously [28] and successfully managed the disinfection of the samples after 5 min and 10 min of immersion [17, 28]. In our study, the disinfection protocol with 0.2% peracetic acid consisted of 10 min immersion, according to the manufacturer’s recommendation for high-level disinfection. Results of this study showed that the use of 0.2% peracetic acid for disinfection with water storage (group 2b) or disinfection and as a storage medium (group 2c) produced no significant horizontal dimensional alteration after 7 days (T168) for Pattern resin groups. For Duralay resin, the use of 0.2% peracetic acid produced a positive significant horizontal dimension alteration when compared to groups 1a and 1e where no disinfection was performed and contraction was observed in all times of storage. Peracetic acid was useful in compensating the shrinkage of the resin for groups 1b (T168), 1c (T24), and 1d (T48).

Superficial changes of the acrylic resins have been evaluated by Chassot et al. [17] who demonstrated that peracetic acid did not affect superficial roughness and color of the resins. However, limited literature is available about the effect of the peracetic acid solution on metallic surfaces [18, 28] and further studies are needed. Additionally, some authors have reported adverse effects of exposure to peracetic acid in higher concentrations (2%) by hospital cleaning staff, such as work-shift eye and upper and lower airway symptoms [29], but no report is available for low concentration as 0.2% peracetic acid solution in dental use.

McDonnell et al. [21] evaluated the accuracy of Pattern and Duralay resin as indexing material after 15 min, 2 h, and 24 h, using the Sheffield 1-screw test as an outcome measure. They found that both acrylic resins were assessed accurately for fit 15 min after polymerization only. The authors recommended that implant assemblies should be invested as soon as possible. Also, according to McDonnell et al. and Dumbrigue et al. [21, 30], AR suffers volumetric shrinkage in the first 24 h. The conclusion of the authors was related only to the polymerization properties of the acrylic resin, considering storage in a dry medium. In our study, AR contraction was observed after 24 h of storage for most of the groups of Duralay resin groups (groups 1a, 1b, 1d, and 1e), except for group 1c (*p* = 0.553), where disinfection and storage were performed with 0.2% peracetic acid. And for Pattern resin groups, contraction after 24 h (T24) was observed for groups 2a (no disinfection and storage in dry medium) and 2c (disinfection and storage in peracetic acid solution). According to our findings, laboratory processing of Pattern resin disinfected with 0.2% peracetic acid should be delayed for more than 48 h at least, to overcome AR shrinkage at 24 h and 48 h, when also stored in 0.2% peracetic solution. If stored in dry medium, laboratory processing should be performed within 24 h (Fig. 4). If stored in water, right after 0.2% peracetic acid disinfection, laboratory processing can be done until 7 days without horizontal dimensional change of the AR.

On the other hand, results obtained by Pattern resin (groups 2b, 2c, and 2d) that were immediately immersed in a 0.2% peracetic solution for 10 min demonstrated a contraction after 24 h (group 2c) and 48 h (groups 2c and 2d), but after 7 days of storage (T168), the horizontal dimension of the specimens was the same as initial measures (T0) for groups 2b (*p* = 1.000) and 2c (*p =* 0.352); therefore, laboratory processing should be delayed. For the Duralay resin group, laboratory processing should be delayed for 7 days for group 1b, 24 h for group 1c, and 48 h for group 1d, when horizontal dimension was the same as the initial dimension (T0).

When comparing the two types of acrylic resin (Pattern × Duralay), most of Pattern resin groups recovered its initial horizontal dimension after 7 days (T168) of storage, regardless of the type of storage (dry × water × peracetic acid) and the use of disinfection solution (no disinfection × disinfection), except for group 2d (disinfection followed by dry storage medium). The Duralay resin group, however, presented no consistent results. Groups not submitted to disinfection (groups 1a and 1e) showed contraction in all storage times, and for those groups submitted to disinfection (groups 1b, 1c, and 1d), only the storage water (group 1b) recovered the horizontal dimension after 7 days.

A couple of limitations must be cited in this study: although one of the purposes of it was to simulate the real clinical steps of indexing IFPDs, it must be considered that a real IFPD would ideally present a larger soldering area, around 9 mm^2^, and therefore, it would need a larger amount of AR for the indexing process. Ultimately, results could evidence differently from the present study. A second limitation was that AR used for indexing IFPDs are subjected to dimensional changes that vary from contraction to expansion, but it is not clear how much of each contribute to the changes observed over the time and if any other factor may play a role in the process. Therefore, further studies should be directed to compare different amounts of resins.

## Conclusion

Within the studied conditions, the use of 0.2% peracetic acid can be safely used as a disinfectant solution regarding the dimensional stability of AR-indexed IFPD until 7 days of storage. Horizontal discrepancies are dependent on acrylic resin type, time, and medium of storage.

## Data Availability

The datasets used and/or analyzed during the current study are available from the corresponding author on reasonable request.

## Abbreviations

AR: Autopolymerized acrylic resin
CoCr: Cobalt-chrome
IFPD: Implant fixed partial denture
SD: Standard deviation
